# Analysis of the regional difference in the number of multi-drug prescriptions and its predictors in Japan, 2015–2018

**DOI:** 10.1186/s13104-021-05787-2

**Published:** 2021-09-20

**Authors:** Tasuku Okui, Jinsang Park

**Affiliations:** 1 Medical Information Center, Kyushu University Hospital, Maidashi 3-1-1 Higashi-ku, Fukuoka City, Fukuoka Prefecture 812-8582 Japan; 2 Department of Pharmaceutical Sciences, International University of Health and Welfare, Fukuoka, Japan

**Keywords:** Japan, Prescriptions, Polypharmacy, Public assistance, Database

## Abstract

**Objective:**

Polypharmacy and multi-drug prescription are major public health problems in Japan, but only a few studies have investigated the regional differences. By revealing regional differences in the multi-drug prescriptions, we can infer regions with high rates of multimorbidity or inappropriate prescribing. This study revealed regional differences in multi-drug prescriptions (the number of simultaneous prescriptions of seven or more internal medicines) and investigated the factors affecting the difference using the National Database of Health Insurance Claims and Specific Health Checkups of Japan data.

**Results:**

The standardized claim ratio (SCR) of the number of multi-drug prescriptions, which corrected the difference in sex and age distribution of prefectures, varied depending on prefectures. A panel data analysis investigating the association between the SCR and explanatory variables (Medical institutions, socioeconomic factors, and physical characteristics of people in prefectures) revealed that the number of public assistance recipients per 1,000 persons was positively and significantly associated with the SCR (Standardized partial regression coefficient = 0.244, p-value = 0.038). In conclusion, regional differences in the number of the multi-drug prescriptions were revealed in Japan, suggesting that public assistance recipients tend to experience multi-drug prescriptions.

**Supplementary Information:**

The online version contains supplementary material available at 10.1186/s13104-021-05787-2.

## Introduction

Polypharmacy is a significant public health issue in many countries [1], including Japan [2]. The age-standardized prevalence rate of polypharmacy (use of five or more oral prescription medications per month) increased from 85.2 to 93.8 per 1,000 persons per month from 2010 to 2016 [2]. Furthermore, the prevalence rate in the population aged 65–79 years is approximately 160 per 1000 people, and the prevalence rate in the population ≥ 80 years is approximately 300 per 1000 people [2]. The prevalence of the use of five or more drugs in the elderly ranged from 26.3% to 39.9% in European countries [3] and 36.8% in the United States [4]. Therefore, the prevalence of polypharmacy in Japan is lower than in the United States, but not particularly lower than in European countries. Polypharmacy can lead to some adverse effects, which tend to occur more frequently as the number of drugs taken increases [5]. The number of prescribed drugs increases with age, and elderly persons particularly need to be given attention in terms of polypharmacy [6].

In order to prevent polypharmacy, some medical fee revisions have been conducted in Japan. Hospitals demand medical fees to the Healthcare bill check and payment organization in the Japanese health insurance system, and prescription fees that the hospital obtains are reduced when seven or more types of internal medicines are prescribed simultaneously to a patient [7]. In addition, the reduction of prescription fees are applied when three or more types of the same kind of psychotropic drugs (one of hypnotics, antidepressants, anxiolytics, and antipsychotics) are prescribed simultaneously [7]. It is important to assess the current status of the multi-drug prescriptions and factors associated with multi-drug prescriptions in Japan using nationally representative data. In other countries, low socioeconomic status as well as multimorbidity are risk factors for polypharmacy [8–11]. In Japan, multimorbidity is a determinant of polypharmacy [12, 13], while only a few studies have examined an association between low socioeconomic characteristics and polypharmacy. National Database of Health Insurance Claims and Specific Health Checkups of Japan (NDB) is a database owned by the Ministry of Health, Labour, and Welfare in Japan [14], and it collects most of claims data in all of Japan under a law. The database contains valuable data for revealing trends of prescriptions, diseases, and medical practices in Japan, and it has been used in previous studies. A part of NDB data is publicly available now as NDB Open data, and studies using them have been conducted recently [15–17]. NDB Open data contains prescription data by prefecture, and it will be useful for revealing regional differences in prescription trends [18]. On the other hand, studies revealing regional differences in multi-drug prescriptions are few in Japan. By revealing regional differences in the multi-drug prescriptions, we could infer regions where the proportion of multimorbidity patients is large or inappropriate prescriptions are conducted. NDB data have been used by the Ministry of Health, Labour, and Welfare for revealing regional differences in proportions of multi-drug prescriptions among all the prescriptions [19]. However, only out-of-prescriptions data gathered from pharmacies have been used [19], and regional differences taking into account in-hospital prescriptions have not been revealed. In addition, we need to take into account the population for each prefecture to compare the number of multi-drug prescriptions among prefectures. Moreover, a study investigating the factors affecting the regional difference has not been conducted in Japan, and the reason for the regional difference is unknown.

In this study, we revealed regional differences in the number of multi-drug prescriptions in Japan and investigated the factorsaffecting the difference.

## Main text

### Materials and methods

In Japan, prescription fees that a medical institution obtains are reduced when seven or more types of internal medicines are simultaneously prescribed to a patient (exceptions are in case of emergency and short administration periods), and the number of multi-drug prescriptions can be determined from claims data. Therefore, medical practice codes of simultaneous prescription of seven or more types of internal medicines were used as an indicator for multi-drug prescriptions (Medical practice code in Japan: 120002610 and 120002710). The number of claims by year, age group, sex, and the number of claims by year for each prefecture in Japan were obtained from the NDB Open data [20]. In addition, we obtained population data by year, age group, and sex from the Basic Resident Register data in Japan [21].

Variables related to medical institutions, socioeconomic factors, and physical characteristics of people in prefectures were used as factors for investigating the association with the number of the simultaneous prescriptions of seven or more types of internal medicines. The variable used and the data source are shown in Additional file 1: Table S1 [20, 22, 23]. The specific health checkups are conducted every year for insured persons and their dependents aged 40–74 years [24]. Data from 2015 to 2018 are publicly available for multi-drug prescriptions, but all the data for each prefecture could only be obtained from 2015 to 2017. Therefore, data from 2015 to 2017 were used for the regression analysis.

For the statistical analysis, we calculated the number of claims for the multi-drug prescription per capita in Japan by sex and age group, and calculated the expected number of the claims for each prefecture taking into account age group and sex-specific population for each prefecture. Then, we calculated the standardized claim ratio (SCR) from the actual number of claims and the expected number of claims as is conducted in previous studies [25, 26]. The mean of SCR is set to be 100 for each year. In addition, a linear mixed-effects model was used in panel data analysis to determine if there is an association between the SCR and the explanatory variables, and information on prefecture was used as a random effect. The outcome (SCR) and the explanatory variables were standardized, and standardized partial regression coefficient (SPRC) and 95% confidence intervals were calculated. A statistically significant difference was judged based on a p-value < 0.05, and two-sided statistical tests were conducted. All statistical analyses were conducted using R 3.6.3 (https://www.r-project.org/). This study does not require the approval of an institutional review committee, as it analyzed publicly available government statistics. Data did not include individual information of patients.

### Results

Table 1 shows the number of the multi-drug prescription per 1000 persons by age group, sex, and year. The number of claims per 1000 persons increased with age, and the number tended to be larger for men than that for women in the middle ages.

**Table 1:** Number of the multi-drug prescriptions per 1000 persons by age group, sex, and year

	Age group																		
Year	0–4	5–9	10–14	15–19	20–24	25–29	30–34	35–39	40–44	45–49	50–54	55–59	60–64	65–69	70–74	75–79	80–84	85–89	> 89
Men																			
2015	5.4	6.6	6.7	7.4	11.3	20.5	31.0	49.0	76.2	120.8	175.4	243.9	329.8	452.2	684.0	965.0	1270.4	1457.6	1353.3
2016	4.5	6.1	6.2	6.8	9.7	17.1	26.4	42.4	68.1	109.8	167.8	232.7	310.0	430.9	624.7	862.9	1149.9	1312.1	1205.2
2017	4.0	5.5	6.4	7.0	9.9	17.3	26.6	41.9	68.4	110.1	168.7	238.3	317.2	435.7	601.7	854.5	1130.5	1300.4	1222.2
2018	3.7	5.3	6.3	6.8	9.0	15.8	24.4	37.5	62.2	100.4	158.0	224.1	296.9	403.6	546.7	766.6	1027.7	1180.6	1114.3
Women																			
2015	4.6	5.4	5.4	8.7	17.4	30.9	41.5	55.3	72.0	92.7	122.2	160.4	213.8	300.1	528.6	855.5	1242.9	1473.9	1243.9
2016	3.9	4.8	5.0	7.5	14.9	26.5	36.0	48.3	65.0	83.2	113.6	149.2	197.0	279.4	467.2	743.7	1097.9	1316.6	1112.0
2017	3.6	4.5	5.1	7.9	15.0	27.0	37.0	49.2	66.5	85.5	114.2	149.9	197.4	277.9	435.4	719.4	1061.9	1288.7	1119.4
2018	3.3	4.4	4.9	7.5	13.8	24.4	33.4	44.5	60.6	78.5	105.4	139.1	182.4	251.8	380.5	625.3	941.8	1148.4	1004.5

Table 2 shows the number of the multi-drug prescription per 1,000 persons and SCR by year and prefecture. The SCR varied depending on prefectures, and the yearly fluctuation of the value was small. The SCR for Nagasaki was the largest every year, and those for Hiroshima and Saga were also large compared with the other prefectures. In contrast, the SCR for Toyama was the smallest every year, and those for Wakayama and Fukui were also relatively small.

**Table 2:** Number of the multi-drug prescriptions per 1000 persons and the SCR by year and prefecture

	Number of the multi-drug prescriptions per 1,000 persons				SCR			
Prefecture	2015	2016	2017	2018	2015	2016	2017	2018
Hokkaido	373.3	341.7	343.3	324.0	126.1	125.3	124.8	127.9
Aomori	363.1	338.8	342.7	320.4	118.0	119.6	119.7	121.4
Iwate	313.7	291.0	296.0	272.8	98.4	99.5	100.7	101.0
Miyagi	312.3	285.9	286.7	266.9	115.6	115.3	114.7	116.0
Akita	413.9	376.0	373.9	349.0	118.7	117.4	115.9	117.8
Yamagata	290.0	271.4	275.1	256.6	89.0	91.1	92.1	93.9
Fukushima	359.9	327.4	326.6	294.8	119.9	119.3	118.3	116.2
Ibaraki	278.3	258.6	259.1	240.9	102.8	103.5	102.8	103.6
Tochigi	271.2	246.2	248.6	229.8	101.1	99.7	99.9	100.2
Gunma	264.3	242.9	245.0	222.1	94.5	94.5	94.6	93.1
Saitama	221.3	206.4	209.3	195.3	89.8	90.4	90.4	91.1
Chiba	203.9	191.5	194.8	178.9	79.8	81.0	81.5	81.0
Tokyo	258.6	237.3	235.5	214.7	107.8	108.3	107.3	107.0
Kanagawa	208.0	194.1	197.0	184.0	84.4	85.3	85.6	86.6
Niigata	256.6	236.4	237.0	219.2	82.6	83.0	82.8	83.5
Toyama	232.8	213.9	213.4	195.9	76.5	76.7	76.1	76.2
Ishikawa	286.2	263.6	263.8	239.0	102.0	102.3	101.7	100.3
Fukui	234.5	214.2	215.0	198.7	80.0	79.8	79.9	80.5
Yamanashi	306.0	277.8	277.2	250.4	104.2	103.0	102.0	100.1
Nagano	280.0	252.0	255.2	232.8	90.6	89.2	90.1	89.7
Gifu	248.3	223.8	228.8	209.0	87.6	86.0	87.2	86.6
Shizuoka	232.8	213.3	214.8	197.5	82.7	82.3	82.3	82.2
Aichi	208.1	192.5	196.0	178.9	85.5	85.9	86.8	86.0
Mie	228.8	206.1	211.7	192.0	80.8	79.2	80.9	79.7
Shiga	226.6	209.3	211.2	189.9	90.2	90.6	90.6	88.5
Kyoto	231.4	214.3	216.5	198.7	83.5	84.0	84.2	84.0
Osaka	259.3	241.4	245.4	221.9	100.4	101.0	101.6	99.8
Hyogo	259.9	238.4	240.1	220.4	96.1	95.6	95.3	95.0
Nara	243.3	229.1	234.4	219.6	85.6	87.1	88.0	89.2
Wakayama	246.8	224.2	227.1	206.0	79.6	78.7	79.1	78.2
Tottori	293.5	271.2	279.2	253.5	94.8	96.0	98.6	97.7
Shimane	369.6	333.8	341.2	320.7	110.1	109.3	111.6	115.3
Okayama	338.6	304.0	300.1	276.3	116.5	114.2	112.2	112.8
Hiroshima	371.8	336.4	333.8	298.8	133.1	131.2	129.4	126.3
Yamaguchi	340.1	308.2	312.3	282.0	105.7	104.5	105.3	103.8
Tokushima	281.6	254.3	262.2	241.0	89.5	88.2	90.7	90.8
Kagawa	336.5	311.5	319.5	305.2	112.8	114.2	116.6	121.6
Ehime	241.1	223.8	230.0	214.2	77.7	78.7	80.5	81.8
Kochi	377.5	345.1	347.1	322.6	113.1	113.0	113.1	114.9
Fukuoka	317.7	291.8	293.8	268.6	120.2	120.3	120.5	120.2
Saga	388.3	353.6	353.4	325.8	134.0	133.2	132.7	133.7
Nagasaki	438.6	399.3	392.6	351.3	142.6	141.5	138.3	134.9
Kumamoto	357.4	324.7	326.2	299.0	118.1	117.4	117.6	117.9
Oita	362.6	332.4	335.1	310.1	116.8	116.9	117.2	118.3
Miyazaki	331.9	306.5	312.0	290.0	108.7	109.4	110.7	112.3
Kagoshima	340.0	303.1	301.8	280.8	109.0	106.4	105.8	107.8
Okinawa	222.4	210.5	213.9	199.6	100.3	103.2	103.9	105.1

*SCR* standardized claim ratio

The Additional file 1: Table S2 shows the median (Q1–Q3) of the explanatory variables used in the linear mixed-effects model.

Table 3 shows the results of the linear mixed-effects model. The absolute value of SPRC indicates the degree of association between the explanatory variable and the outcome, and the larger the SPRC, the stronger is the association. The linear mixed-effects model assumes that the outcome variables follow a normal distribution, thereby confirming that the SCR distribution does not deviate significantly from the normal distribution.

**Table 3:** The results of linear mixed effects model

Variable	SPRC (95% CI)	p-value
Medical institutions		
Number of hospitals per 100,000 persons	0.190 (− 0.095, 0.477)	0.216
Number of medical clinics per 100,000 persons	− 0.075 (− 0.294, 0.144)	0.522
Number of pharmacies per 100,000 persons	0.124 (− 0.024, 0.285)	0.106
Socioeconomic factors		
Population density (person per hectare)	0.060 (− 0.268, 0.389)	0.733
Proportion of non-Japanese persons	0.011 (− 0.189, 0.203)	0.914
Number of public assistance recipients per 1000 persons	0.244 (0.027, 0.461)	0.038
Taxable income per capita (unit: 1000 yen)	− 0.045 (− 0.318, 0.222)	0.752
Financial capability index	− 0.098 (− 0.343, 0.159)	0.458
Physical characteristics		
Proportion of persons whose HbA1c (NGSP) ≥ 6.5 (%)	0.018 (− 0.037, 0.073)	0.546
Proportion of persons whose systolic BP ≥ 140 (mmHg)	0.020 (− 0.062, 0.100)	0.640
Proportion of persons whose BMI ≥ 25 (kg/m^2^)	0.028 (− 0.106, 0.163)	0.697
Proportion of persons whose triglycerides ≥ 150 (mg/dl)	− 0.021 (− 0.158, 0.111)	0.763
Proportion of elderly persons requiring support or nursing care	0.026 (− 0.079, 0.133)	0.646

*SPRC* standardized partial regression coefficient,* CI* confidence intervals,* NGSP* National Glycohemoglobin Standardization Program,* BP* blood pressure,* BMI* body mass index

As a result, the number of public assistance recipients per 1000 persons was positively associated with the SCR (SPRC = 0.244, p-value = 0.038), and the absolute value of the SPRC was also the largest among the explanatory variables. On the other hand, the physical characteristics were not associated with the outcome.

### Discussion

We showed regional differences in multi-drug prescriptions in Japan and identified a factor associated with the difference. The number of multi-drug prescriptions in a prefecture was associated with the number of recipients of public assistance in the prefecture. Here we briefly discuss the possible reasons for this association.

Persons with low income have been shown to be related to multimorbidity. Multimorbidity is closely related to polypharmacy [27, 28], and is considered to be a cause of multi-drug prescription. There are many studies that show the association between multimorbidity and low socioeconomic status [29, 30]. Low socioeconomic status is associated with several causes of morbidity in Japan, such as obesity, hypertension, diabetes complications, and psychological distress [31–34]. It is also related to health behaviors, such as smoking and heavy alcohol drinking [35]. Moreover, low socioeconomic status leads to lower utilization of medical care [36, 37], and there is a possibility that delay of early treatment and early detection of diseases can lead to multimorbidity.

In addition, it is considered that patients with multimorbidity or psychiatric diseases tend to become recipients of public assistance because these patients have difficulties in working [38, 39]. The proportion of lifestyle-related diseases are known to be higher in recipients of public assistance compared with the general population [40]. In addition, the prevalence of patients with a psychiatric disease is higher in recipients of public assistance [41], and psychiatric diseases and lifestyle-related diseases, such as metabolic syndromes, have been reported to be closely related [42]. Besides, it is known that a certain number of recipients of public assistance frequently consult a medical institution [43], possibly because hospital visits are free of charge for recipients of public assistance. These frequent visits to a hospital might be somehow related to the multi-drug prescriptions.

On the other hand, physical characteristics related to lifestyle-related diseases were not associated with the outcome. It is known that obesity or other lifestyle-related diseases are associated with polypharmacy [44–46]. Participation in specific health checkups is not mandatory in Japan [47]; thus, there is a possibility that people with low health status tended not to participate in the checkups. In addition, uninsured persons do not participate in the checkups. Therefore, there is a possibility that the participation rate per capita varied depending on prefectures, which might have affected the results regarding the physical characteristics.

This study indicated a possibility that public assistance recipients tend to experience multi-drug prescriptions and fall into polypharmacy. It is considered that multimorbidity patients or psychiatric patients tend to become recipients of public assistance. However, for some public assistance recipients, there are cases of excessive multi-drug prescriptions. Therefore, it is necessary for medical practitioners and social workers to recheck the number of medicines taken by public assistance recipients and their consultation behaviors to medical institutions. In addition, putting restrictions on frequent visits to medical institutions might be needed for some recipients of public assistance, in order to prevent unnecessary multi-drug prescriptions.

### Limitations

There are some limitations to this study. First, the classification of claims data by the prefectures was conducted based on the place of medical institutions, and some differences between a prefecture of medical institutions where multi-drug prescriptions were conducted and a prefecture where a patient with multi-drug prescriptions lives may have existed. Secondly, this is an ecological study, and there was a possibility that the association would not be evident upon analysis of individual data. A further study investigating an association between the recipient of public assistance and multi-drug prescriptions using individual data is needed for verification. Thirdly, we could not reveal the types of drugs for the multi-drug prescriptions and the diseases of patients to whom multi-drugs prescriptions were given. By revealing these characteristics, we could better understand the reason for the regional differences. Finally, some unobserved confounding factors might explain the association between the number of the recipients of public assistance and the multi-drug prescriptions. Factors, such as proportion of persons having psychological distress, proportion of patients with chronic diseases, frailty, and educational level need to be taken into account in a future research [48–50]. On the other hand, this study used the NDB data in Japan, and the results of this study represents trends in all of Japan and are generalizable to all Japnanese people.

## Supplementary Information

**Additional file 1**: **Table S1**. Variables used in the analysis. **Table S2**. Median (Q1-Q3) of the explanatory variables used in the linear mixed effects model.

## Data Availability

The data used in this study can be obtained from websites of government statistics in Japan (The Survey of population, demographics, and household number based on the basic resident register. [cited 17 July 2021]. Available from: https://www.e-stat.go.jp/stat-search/files?page=1&toukei=00200241&tstat=000001039591; e-Stat General counter for government statistics. [cited 17 July 2021]. Available from: https://www.e-stat.go.jp/regional-statistics/ssdsview; NDB Open data. [cited 17 July 2021]. Available from: https://www.mhlw.go.jp/stf/seisakunitsuite/bunya/0000177182.html.;Data of the Report on the Status of the Long-term Care. [cited 19 August 2021]. Available from: https://www.e-stat.go.jp/stat-search/files?page=1&toukei=00450351&tstat=000001031648.

## Abbreviations

SPRC: Standardized partial regression coefficient
CI: Confidence intervals
NGSP: National Glycohemoglobin Standardization Program
BP: Blood pressure
BMI: Body mass index
SCR: Standardized claim ratio (SCR)
NDB: National Database of Health Insurance Claims and Specific Health Checkups of Japan (NDB
