# Supplementary material for: Analysis of the regional difference in the number of multi-drug prescriptions and its predictors in Japan, 2015–2018
Source: BMC Res Notes. 2021 Sep 20;14:367. doi: 10.1186/s13104-021-05787-2 (PMC8454144; doi:10.1186/s13104-021-05787-2)
Supplement: Supplementary file 1 — Additional file 1: Table S1. Variables used in the analysis. Table S2. Median (Q1-Q3) of the explanatory variables used in the linear mixed effects model. [file 13104_2021_5787_MOESM1_ESM.docx]

Additional file 1

Table S1. Variables used in the analysis

| Variable | Data source |
| --- | --- |
| Medical institutions |  |
| Number of hospitals per 100,000 persons | The Survey of Medical Institutions and the Basic Resident Register data [22] |
| Number of medical clinics per 100,000 persons | The Survey of Medical Institutions and the Basic Resident Register data [22] |
| Number of pharmacies per 100,000 persons | The Report on Public Health Administration and Services and the Basic Resident Register data [22] |
| Socioeconomic factors |  |
| Population density (person per hectare) | The Survey on Areas by municipalities and the Basic Resident Register data [22] |
| Proportion of non-Japanese persons | The Basic Resident Register data [22] |
| Number of public assistance recipients per 1,000 persons | The National Survey on Public Assistance Recipients and the Basic Resident Register data [22] |
| Taxable income per capita (Unit:1,000 yen) | The Status of Taxation for Municipal Tax and the Basic Resident Register data [22] |
| Financial capability index | The Survey on Local Financial State [22] |
| Physical characteristics |  |
| Proportion of persons whose HbA1c (NGSP) ≥ 6.5 (%) | The Specific Health Checkups [20] |
| Proportion of persons whose systolic BP ≥ 140 (mmHg) | The Specific Health Checkups [20] |
| Proportion of persons whose BMI ≥ 25 (kg/m^2^) | The Specific Health Checkups [20] |
| Proportion of persons whose triglycerides ≥150 (mg/dl) | The Specific Health Checkups [20] |
| Proportion of elderly persons requiring support or nursing care | The Report on the Status of the Long-term Care [23] |
| NGSP, National Glycohemoglobin Standardization Program; BP, blood pressure; BMI, body mass index | |

Table S2. Median (Q1-Q3) of the explanatory variables used in the linear mixed effects model

|  | Year | | |
| --- | --- | --- | --- |
| Variable | 2015 (n=47) | 2016 (n=47) | 2017 (n=47) |
| Medical institutions |  |  |  |
| Number of hospitals per 100,000 persons | 7.1 (5.9 - 9.7) | 7.2 (5.8 - 9.6) | 7.3 (5.9 - 9.6) |
| Number of medical clinics per 100,000 persons | 81.1 (72.9 - 89.3) | 81.3 (73.0 - 89.9) | 81.2 (73.2 - 89.7) |
| Number of pharmacies per 100,000 persons | 46.6 (43.5 - 51.8) | 47.1 (43.8 - 52.1) | 47.8 (44.1 - 52.6) |
| Socioeconomic factors |  |  |  |
| Population density (person per hectare) | 2.7 (1.8 - 4.9) | 2.7 (1.8 - 4.8) | 2.7 (1.8 - 4.8) |
| Proportion of non-Japanese persons | 1.0 (0.6 - 1.8) | 1.1 (0.6 - 1.9) | 1.2 (0.7 - 2.0) |
| Number of public assistance recipients per 1,000 persons | 13.4 (9.0 - 18.3) | 13.4 (9.0 - 18.1) | 13.4 (9.1 - 18.0) |
| Taxable income per capita (Unit:1,000 yen) | 1275.6 (1112.5 - 1363.4) | 1306.5 (1150.5 - 1396.5) | 1334.6 (1181.2 - 1426.0) |
| Financial capability index | 0.5 (0.3 - 0.6) | 0.5 (0.3 - 0.6) | 0.5 (0.4 - 0.6) |
| Physical characteristics |  |  |  |
| Proportion of persons whose HbA1c (NGSP) ≥ 6.5 (%) | 6.9 (6.5 - 7.4) | 7.1 (6.6 - 7.6) | 7.3 (6.7 - 7.9) |
| Proportion of persons whose systolic BP ≥ 140 (mmHg) | 17.7 (16.7 - 18.9) | 17.9 (16.6 - 19.0) | 17.9 (17.0 - 19.1) |
| Proportion of persons whose BMI ≥ 25 (kg/m^2^) | 26.1 (25.1 - 28.1) | 26.6 (25.6 - 28.7) | 27.4 (26.2 - 29.5) |
| Proportion of persons whose triglycerides ≥150 (mg/dl) | 21.0 (19.8 - 21.4) | 21.0 (20.0 - 21.6) | 21.0 (20.0 - 21.6) |
| Proportion of elderly persons requiring support or nursing care | 18.8 (17.5 - 19.4) | 18.5 (17.3 - 19.5) | 18.4 (17.0 - 19.5) |
| NGSP, National Glycohemoglobin Standardization Program; BP, blood pressure; BMI, body mass index | | |  |
